# Proteomic Analysis of Early Mid-Trimester Amniotic Fluid Does Not Predict Spontaneous Preterm Delivery

**DOI:** 10.1371/journal.pone.0155164

**Published:** 2016-05-23

**Authors:** Maria Hallingström, Juraj Lenco, Marie Vajrychova, Marek Link, Vojtech Tambor, Victor Liman, Maria Bullarbo, Staffan Nilsson, Panagiotis Tsiartas, Teresa Cobo, Marian Kacerovsky, Bo Jacobsson

**Affiliations:** 1 Department of Obstetrics and Gynecology, Institute of Clinical Sciences, Sahlgrenska Academy, University of Gothenburg, Gothenburg, Sweden; 2 Department of Molecular Pathology and Biology, Faculty of Military Health Sciences, University of Defense, Hradec Kralove, Czech Republic; 3 Biomedical Research Center, University Hospital Hradec Kralove, Hradec Kralove, Czech Republic; 4 Department of Neurochemistry, Sahlgrenska University Hospital, Gothenburg, Sweden; 5 Mathematical Sciences, Chalmers University of Technology, Gothenburg, Sweden; 6 Department of Obstetrics and Gynecology, Sahlgrenska University Hospital, Gothenburg, Sweden; 7 BCNatal—Barcelona Center for Maternal-Fetal and Neonatal Medicine (Hospital Clínic and Hospital Sant Joan de Deu), Fetal i+D Fetal Medicine Research Center, Institut d’Investigacions Biomèdiques Agustí Pi I Sunyer (IDIBAPS), University of Barcelona, Barcelona, Spain; 8 Centro de Investigaciones Biomedicas en Enfermedades Raras (CIBER-ER), Barcelona, Spain; 9 Department of Obstetrics and Gynecology, Charles University in Prague, Faculty of Medicine in Hradec Kralove, Hradec Kralove, Czech Republic; 10 Department of Genetics and Bioinformatics, Area of Health Data and Digitalisation, Institute of Public Health, Oslo, Norway; University of Southern California, UNITED STATES

## Abstract

**Objective:**

The aim of this study was to identify early proteomic biomarkers of spontaneous preterm delivery (PTD) in mid-trimester amniotic fluid from asymptomatic women.

**Methods:**

This is a case-cohort study. Amniotic fluid from mid-trimester genetic amniocentesis (14–19 weeks of gestation) was collected from 2008 to 2011. The analysis was conducted in 24 healthy women with subsequent spontaneous PTD (cases) and 40 randomly selected healthy women delivering at term (controls). An exploratory phase with proteomics analysis of pooled samples was followed by a verification phase with ELISA of individual case and control samples.

**Results:**

The median (interquartile range (IQR: 25^th^; 75^th^ percentiles) gestational age at delivery was 35+5 (33+6–36+6) weeks in women with spontaneous PTD and 40+0 (39+1–40+5) weeks in women who delivered at term. In the exploratory phase, the most pronounced differences were found in C-reactive protein (CRP) levels, that were approximately two-fold higher in the pooled case samples than in the pooled control samples. However, we could not verify these differences with ELISA. The median (25^th^; 75^th^ IQR) CRP level was 95.2 ng/mL (64.3; 163.5) in women with spontaneous PTD and 86.0 ng/mL (51.2; 145.8) in women delivering at term (*p* = 0.37; t-test).

**Conclusions:**

Proteomic analysis with mass spectrometry of mid-trimester amniotic fluid suggests CRP as a potential marker of spontaneous preterm delivery, but this prognostic potential was not verified with ELISA.

## Introduction

Preterm delivery (PTD) is a current global concern in obstetric and neonatal care [1, 2]. It is related to short- and long-term morbidity in neonates [3] and is a leading cause of child death worldwide. Approximately two-thirds of PTDs are spontaneous [4]. The etiology behind spontaneous PTD is complex, and understanding of the sequence and timing of events preceding the condition is incomplete [5].

Proteomics, one of the most promising platforms for biomarker detection, provides insight into the basic biological mechanisms involved in a condition. It constitutes an alternative unbiased approach, compared to the widely used hypothesis-based biomarker discovery approach [6, 7]. Previous studies have explored the maternal, fetal [8–13] and amniotic fluid proteomes and their associations with intra-amniotic inflammation, intra-amniotic infection and PTD in women with preterm labor or preterm prelabor rupture of membranes [8, 10, 11, 14–21]. However, there are only a few published studies exploring the potential of proteomics early in pregnancy, before onset of symptoms. To the best of our knowledge, Fotopoulou et al. [15] are the only researchers who have investigated the protein composition in mid-trimester amniotic fluid in relation to spontaneous PTD, using mass spectrometry profiling. However, their results have not been verified, as is considered mandatory for proteomic analysis [22], representing a considerable limitation.

Due to the complexity and incompletely identified pathophysiological pathways of spontaneous PTD, clinical applications of proteomic technology are still in the early stages [5]. Moreover, the translation of proteomic knowledge into the clinical setting requires verification with an easier, rapid, cost-effective method.

Identification of early prognostic or diagnostic biomarkers, before onset of clinical symptoms, is important. The main aim of this study was therefore to explore potential early biomarkers for spontaneous PTD during the mid-trimester of pregnancy in an exploratory proteomics phase with a pooled sample strategy, utilizing liquid chromatography-tandem mass spectrometry (LC-MS/MS). The second aim was to verify the difference in candidate protein levels found between cases and controls, using ELISA in individual samples from the same cohort.

## Materials and Methods

### Study design

This study was a case-cohort study of women, aged over 18, who underwent a mid-trimester transabdominal genetic amniocentesis at 14–19 weeks of gestation in a viable singleton pregnancy. Amniocentesis indications were advanced maternal age, anxiety, abnormal first-trimester combined screening or family history of chromosomal abnormalities or genetic diseases. Age under 18 years, multiple pregnancy, positive HIV or hepatitis B test and known or suspected fetal malformation were exclusion criteria. Women who could not understand the written and oral information in Swedish, who declined participation or from whom insufficient amniotic fluid was retrieved at amniocentesis were also excluded.

After medical review, women with chronic diseases (e.g. severe rheumatism, hypo- or hyperthyroidism, severe asthma, diabetes mellitus, hypertension, multiple sclerosis, hereditary chromosomal defects, vitamin D deficiency and severe neurological disorders) were excluded from analysis.

Women with a subsequent spontaneous PTD were compared with women who delivered at term. For the sake of homogeneity, the term delivery group was limited to women giving birth at gestational weeks 38+0 to 41+6, among whom a random selection was made in order to constitute the control group. Medical records were scrutinized at inclusion and after delivery.

### Sample collection

An additional 3 mL of amniotic fluid was collected during mid-trimester genetic transabdominal amniocentesis and immediately stored at 4-8°C. The samples were centrifuged for 20 minutes at 12°000 *g*, at 4°C. The supernatant was separated from the pellet and divided into aliquots that were frozen and stored at -80°C. All samples were handled according to a standardized protocol at the same laboratory.

### Exploratory proteomics phase: sample preparation and LC-MS/MS analysis

**Fig 1:**
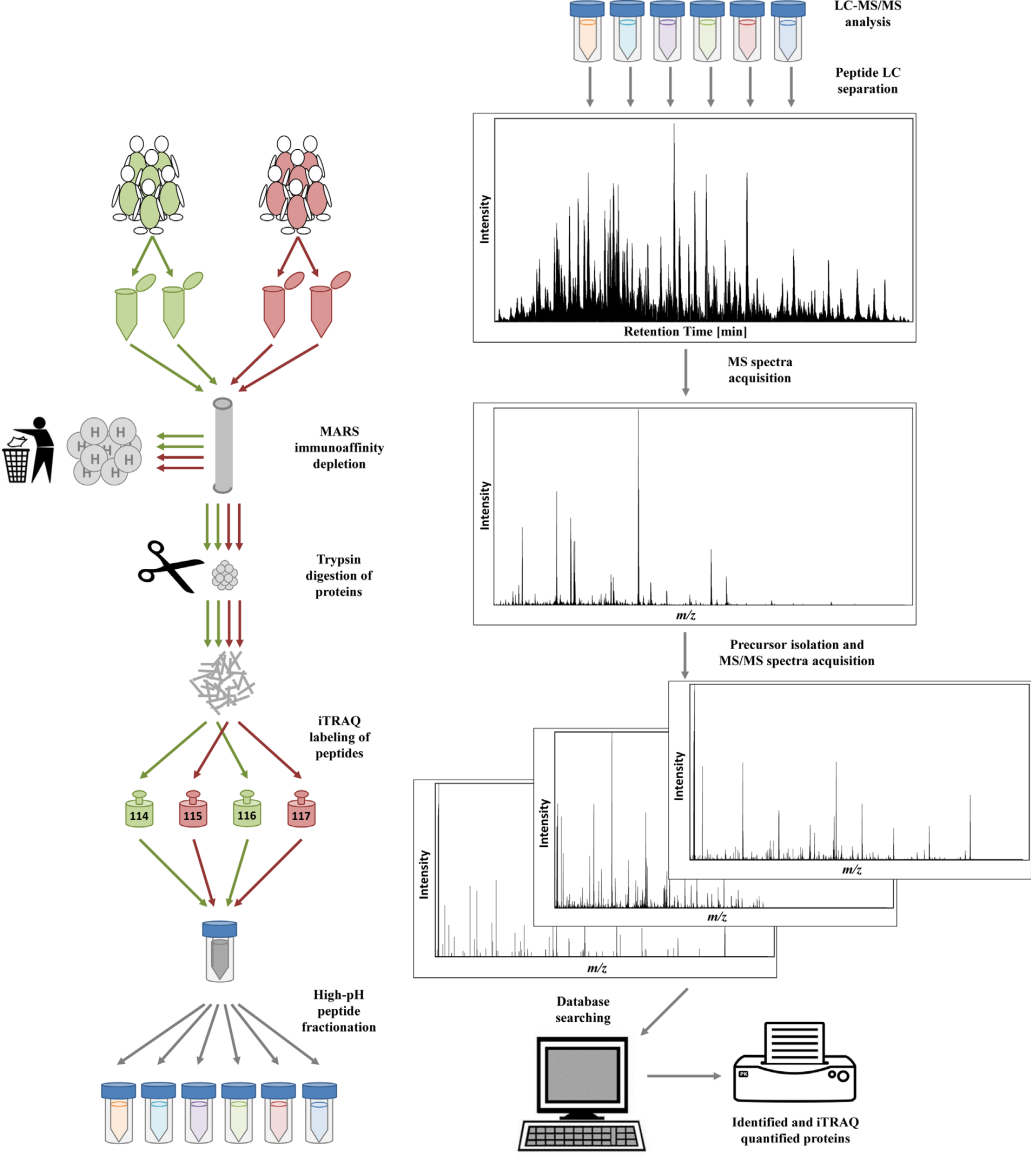
Graphic presentation of the methodology of sample processing and LC-MS/MS analysis of pooled case and control samples.

For the exploratory proteomic analysis, pooled amniotic fluid supernatant samples from cases and controls were prepared according to the approach reported by Tambor et al. [23]. Briefly, the samples were supplemented with protease inhibitors and filtered. An equal amount of protein was taken from each sample to create a pooled representative control sample and a representative case sample; both were created in duplicate. The most abundant proteins in the samples were removed using a MARS Hu-14 immunoaffinity column (Agilent, Palo Alto, CA). The proteins were then reduced with tris (2-carboxyethyl) phosphine hydrochloride and the thiol groups were blocked with methyl methane thiosulfonate. Proteins were digested with trypsin at 37°C overnight. The peptides in the representative control samples were labeled with isobaric tags for relative and absolute quantification using iTRAQ 114 and 116 tags, while representative case samples were labeled with iTRAQ 115 and 117 tags (AB Sciex, Foster City, CA). High-pH reversed-phase chromatography was then employed to fractionate the peptides into six 2-minute fractions. An UltiMate 3000 RSLCnano system hyphenated to a Q-Exactive mass spectrometer (Thermo Scientific, Bremen, Germany) was used for protein identification and quantification, using the full MS/Top10 experimental setup. The recorded HCD fragmentation spectra were evaluated in Proteome Discoverer software (Thermo Scientific), using MASCOT (Matrix Science, London, UK) against the human UniProt database. When it came to quantification, only proteins up to 1% FDR threshold were reported and only proteins quantified with at least three peptides were evaluated. The most significantly regulated proteins were found by global ranking method [24]. For both duplicates, 25 proteins were evaluated from the top and from the bottom of list of proteins ranked based on the relative quantitative change (Fig 1). Further specific details regarding the exploratory proteomics phase can be found in the supporting materials and methods (S1 File).

### Verification of amniotic fluid CRP levels by ELISA immunoassay

Amniotic fluid C-reactive protein (CRP) levels were determined in individual case samples and control samples with commercially available ELISA kits (Quantikine ELISA, R & D Systems, Minneapolis, MN). All samples were initially diluted 1/100, but if the subsequent absorbance was below the detection limit for the assay, a 1/10 dilution was used instead. The detection range of the assay was 0.78–50 ng/mL, but dilutions accommodated a practical detection range of 7.8–5000 ng/mL. All of the samples were run in duplicate. The interassay coefficient of variation (CV) was <15%, and the intraassay CV was <5% on all three plates. If duplicates varied more than 15%, the samples were rerun. The resulting absorbance was read at 450 nm (Multiskan FC Microplate Photometer).

### Ethics statement

This study was approved by the Central Ethical Review Board at the University of Gothenburg, Sweden (Dnr. Ö 639–03, T 318–08, T 694–11, T 958–11). Written informed consent was obtained from all participants.

### Statistical analyses

Continuous variables were compared using a nonparametric Mann-Whitney *U* Test and presented as median and interquartile range (IQR: 25^th^; 75^th^ percentiles). Categorical variables were compared using Fisher’s Exact Test and presented as the number (%). Differences were considered statistically significant at *p* < 0.05 using a two-sided alternative hypothesis. All statistical analyses were performed with SPSS 20.0 for Windows XP OS (SPSS Inc., USA).

## Results

### Characteristics of the entire study group

From September 2008 to September 2011, 1546 women underwent mid-trimester amniocentesis at Sahlgrenska University Hospital/Östra, Gothenburg, Sweden. Of this population, 426 women did not fulfill the strict inclusion criteria and 495 were excluded, the majority of whom because they declined participation. An additional 18 women were excluded from analysis; twelve requested a termination of pregnancy due to chromosomal abnormality and six were lost to follow-up.

The remaining women (*n* = 607) were included in the study. Their medical records were scrutinized at inclusion and after delivery. PTD occurred in 6.3% (38/607), of which 4.8% (29/607) were spontaneous PTD and 1.5% (9/607) were iatrogenic PTD. Of the group with spontaneous PTD, five women were excluded due to hypothyroidism, severe asthma, bicornuate uterus, type 1 diabetes, translocation and/or discolored amniotic fluid at sampling. Therefore, the remaining women with spontaneous PTD (*n* = 24) constitute the cases in this study.

**Fig 2:**
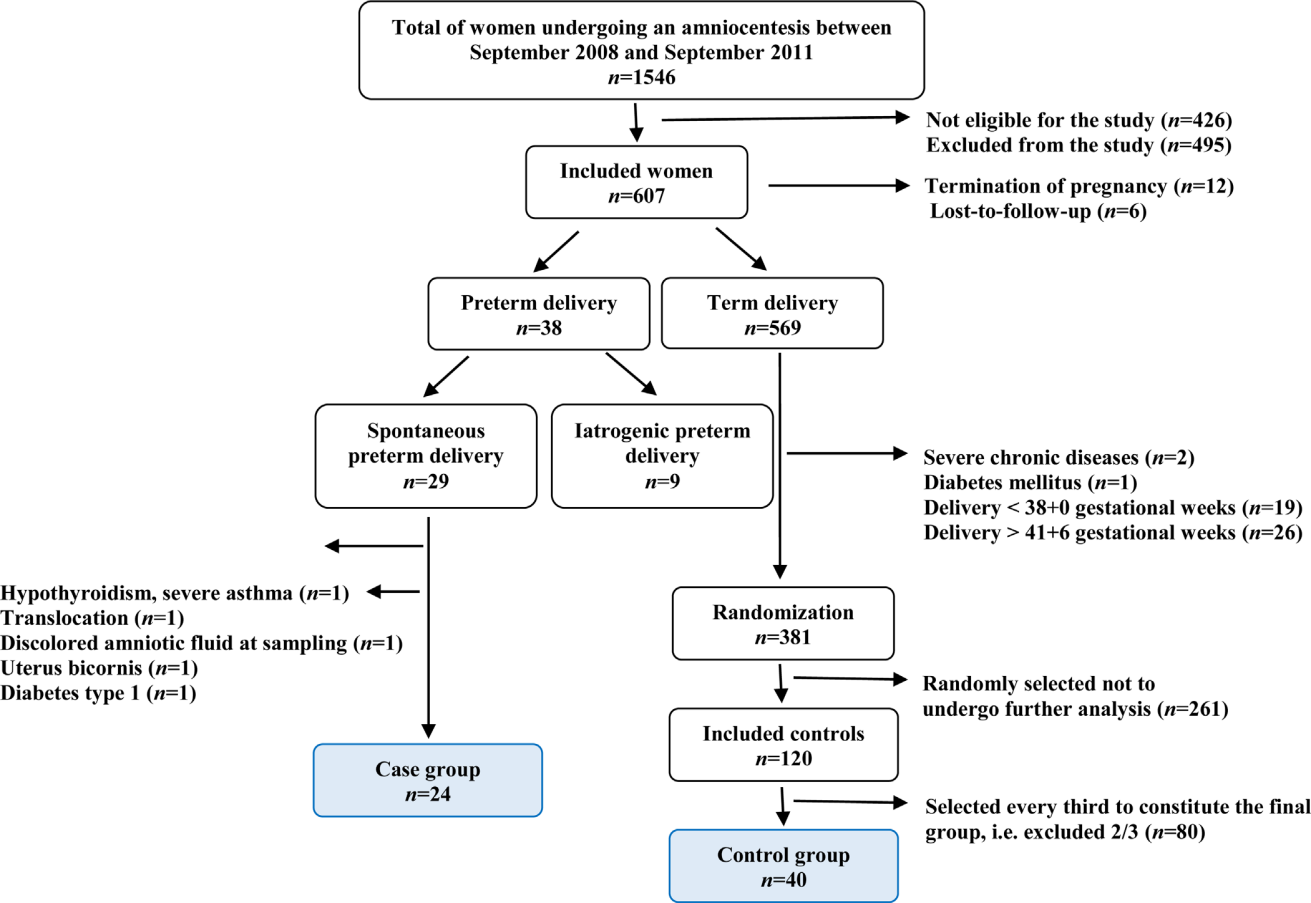
Flow chart showing selection of study participants.

Of the 569 women who delivered at ≥37 gestational weeks, 381 were healthy and gave birth at gestational week 38+0 to 41+6. Forty were randomly selected and they constituted the final control group in this study (Fig 2).

The respective maternal and neonatal characteristics of the spontaneous PTD and term delivery groups are presented in Table 1. There were no significant differences between the groups, except for the obvious difference in birth weight.

**Table 1:** Maternal and neonatal characteristics in the group of women with spontaneous preterm delivery and the group of women with term delivery.

Variable	Spontaneous preterm delivery (*n* = 24)	Term delivery (n = 40)	*p*
Gestational age at delivery (weeks+days)	35+5 (33+6–36+6)	40+0 (39+1–40+5)	
Maternal age at sampling (years)	37 (36–40)	36 (35–38)	0.69
Nulliparous	9 (37.5%)	12 (30.0%)	0.59
IVF	4 (16.7%)	1 (2.5%)	0.06
Maternal BMI at first prenatal visit	24.6 (23.6–26.9)	23.5 (21.2–26.3)	0.23
Smoking at first prenatal visit	2 (8.3%)	2 (5.0%)	0.63
Previous preterm delivery	3 (12.5%)	1 (2.5%)	0.14
Gestational age at sampling (weeks+days)	15+5 (15+2–16+3)	15+5 (15+2–16+2)	0.69
Mode of delivery			
Vaginal delivery	21 (87.5%)	28 (70.0%)	0.14
Caesarean section	3 (12.5%)	8 (20.0%)	0.51
Vacuum extraction	0 (0%)	4 (10.0%)	0.29
Birth weight (grams)	2505 (2378–2848)	3530 (3215–3713)	**<0.0001**
Gender			0.18
Male	9 (37.5%)	21 (52.5%)	
Female	15 (62.5%)	19 (47.5%)	
Apgar score < 7 at 5 min	0 (0%)	1 (2.6%)	0.62

Continuous variables were compared using a nonparametric Mann-Whitney *U* Test and presented as the median (interquartile range). Categorical variables were compared using Fisher’s Exact Test and presented as the number (%).

### Exploratory proteomics phase: LC-MS/MS analysis

In the exploratory phase, a total of 77411 spectra were searched, and an amino sequence was assigned in 24641 spectra (1% false discovery rate (FDR)), resulting in the identification of 7267 distinct peptides and 1088 protein groups. Of the protein groups, 594 were quantified with at least three peptides. Of these, 17 proteins were reproducibly upregulated (FDR 0.029) and 19 proteins were downregulated (FDR 0.026), as shown in S1 and S2 Tables. Five upregulated and five downregulated proteins were among top 10 dysregulated proteins independently on the direction of the change (Table 2). The most pronounced difference between levels in the pooled control and pooled case samples was found in the case of CRP (P02741, CRP), which was selected for further verification with ELISA.

**Table 2:** The ten most dysregulated proteins in both duplicates (115/114 and 117/116) from the exploratory proteomics analysis, where 115 and 117 represent the channels for the cases and 114 and 116 represent the channels for the controls.

Prot. Acc. #	Gene	Description	115/114	117/116
P02741	CRP	C-reactive protein	2.27	2.30
P60174	TPI1	Triosephosphate isomerase	2.26	1.95
A8K7I4	CLCA1	Calcium-activated chloride channel regulator 1	1.75	1.98
P40925	MDH1	Malate dehydrogenase, cytoplasmic	1.86	1.63
P01037	CST1	Cystatin-SN	1.46	1.51
P02042	HBD	Hemoglobin subunit delta	0.56	0.52
P69905	HBA1	Hemoglobin subunit alpha	0.60	0.51
P68871	HBB	Hemoglobin subunit beta	0.61	0.52
P09466	PAEP	Glycodelin	0.61	0.59
P49913	CAMP	Cathelicidin antimicrobial peptide	0.65	0.71

As reported in the table, five upregulated and five downregulated proteins were among top 10 dysregulated proteins independently on the direction of the change. The level of CRP was roughly two-fold higher in the pooled samples from cases (115, 117), compared to the pooled samples from controls (114, 116).

### Verification of amniotic fluid CRP level difference from the exploratory phase

**Fig 3:**
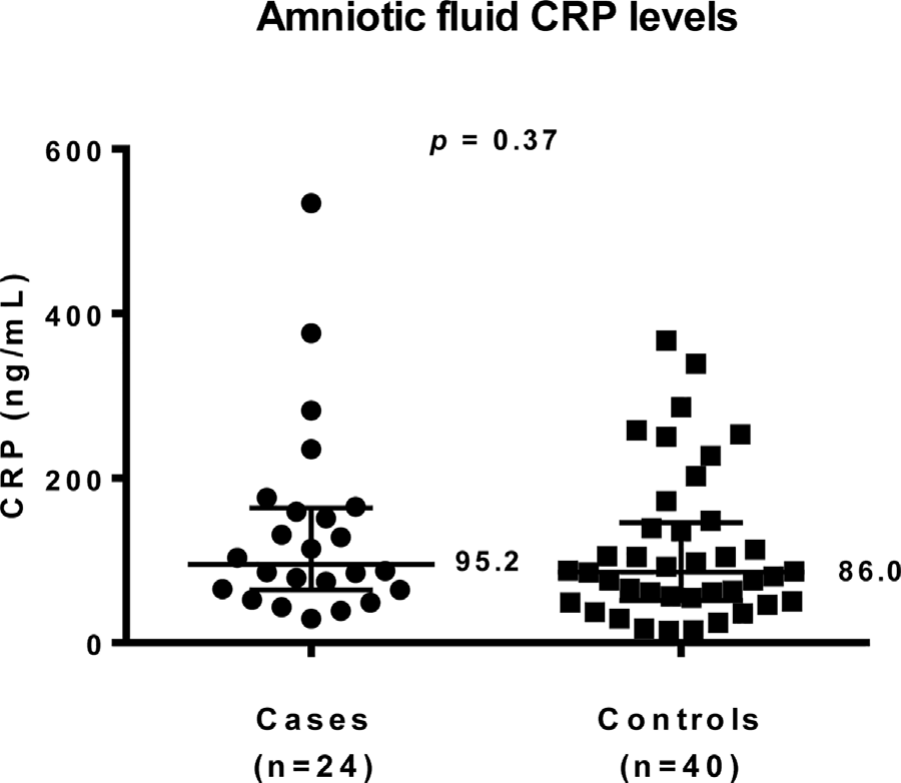
A two-column scatter graph of median (IQR: 25^th^; 75^th^ percentiles) amniotic fluid CRP levels in the groups.

In the exploratory proteomics analysis, CRP levels were approximately two-fold higher in cases than in controls in both duplicates. We attempted to verify this difference in the same women, but with an individual ELISA analysis of each case and control sample. The median (IQR: 25^th^; 75^th^ percentiles) CRP level (ng/mL) in the spontaneous PTD group was 95.2 (64.3; 163.5) compared to 86.0 (51.2; 145.8) in the term delivery group, a difference that was not significant (*p* = 0.37) (Fig 3).

## Discussion

The exploratory proteomics phase of this study, analyzing pooled samples from mid-trimester amniotic fluid in asymptomatic women, revealed rather few dysregulated proteins. CRP was selected for verification since it had the most pronounced difference in levels between the control and case groups. Furthermore, it was the subject of previous publications and was readily available as a commercial human ELISA test. However, the case-control CRP level difference we initially found with proteomics, and the related prognostic potential, was not verified on subsequent individual analysis with ELISA.

CRP is a very well described marker of systemic inflammation [25] and it is associated with both PTD and preterm prelabor rupture of membranes [26, 27]. Several studies have previously performed targeted analyses of CRP in mid-trimester amniotic fluid [26, 28–31], with contradictory results. While three studies [28, 29, 31] did not find any significant differences between amniotic fluid CRP levels in cases of spontaneous PTD and term deliveries, Ozer et al. [30] and Ghezzi et al. [26] did find significant differences. These conflicting results highlight our effort to analyze this protein using a different but complementary approach.

The mid-trimester amniotic fluid proteome has been explored in relation to spontaneous PTD in twins [32] but there is, to our knowledge, only one published study exploring mid-trimester amniotic fluid as a predictor of spontaneous PTD in singletons. Fotopoulou et al. [15] investigated a small group of women with both spontaneous PTD and term deliveries. Using a combination of mass spectrometry and different solid-phase chromatography protein chips, they identified seven clusters that differed significantly when women with a subsequent spontaneous PTD were compared with women who delivered at term. However, the individual proteins/peptides could not be identified by SELDI TOF analysis.

A major strength of our study is the verification phase, otherwise frequently neglected. The standardized protocol for sample handling and the strict selection criteria for the case and control groups are additional strengths. Furthermore, this study had a relatively large cohort of healthy, asymptomatic women with subsequent spontaneous PTD and a very low lost-to-follow-up rate.

An important limitation of the study was that one single biomarker (CRP) was selected for further verification. We refrained from considering other potential biomarkers that had, in contrast to CRP, not been the objects of previous published mid-trimester studies. A pooling strategy provides an initial proteomic evaluation, albeit not as expensive or time-consuming as proteomic analysis of each and every individual sample. However, the pooled sample strategy inevitably has certain limitations. One such limitation is the absence of estimates of individual variability, required to facilitate significance testing. We hypothesized that the discrepancy between the exploratory phase and the verification stage might have been caused by the fact that some samples may have contained extremely low/high analyte levels (outliers) and may thus have shifted levels in the entire pool. The key prerequisite if samples are pooled is thus results verification in individual samples with an alternative method, which we attempted using ELISA. Finally, another limitation of this study might be that the spontaneous PTD group consisted of women with both preterm labor and preterm prelabor rupture of membranes; the respective phenotypes may differ somewhat.

## Conclusions

The exploratory proteomic phase of the experiment revealed six dysregulated proteins, of which CRP levels were approximately two-fold higher among women with spontaneous PTD, compared to controls. However, when individual cases and controls were examined with ELISA, there was no significant difference in CRP levels between the case and control groups. This finding is interesting in light of how extensively this test is used, and confirms the current ambiguity concerning its status. Further proteomics studies are needed, on individual samples and with similar strict criteria for the groups as in this study.

## Supporting Information

S1 File Materials and Methods. (DOCX)

S1 Table The complete list of the 17 proteins that were reproducibly upregulated where 115 and 117 represent the channels for the cases and 114 and 116 represent the channels for the controls. The proteins are ranked according to the absolute value of log2 of average of 115/114 and 117/116 ratio.
(DOCX)

S2 Table The complete list of the 19 proteins that were downregulated where 115 and 117 represent the channels for the cases and 114 and 116 represent the channels for the controls. The proteins are ranked according to the absolute value of log2 of average of 115/114 and 117/116 ratio.
(DOCX)
