# Supplementary material for: Proteomic Analysis of Early Mid-Trimester Amniotic Fluid Does Not Predict Spontaneous Preterm Delivery
Source: PLoS One. 2016 May 23;11(5):e0155164. doi: 10.1371/journal.pone.0155164 (PMC4876998; doi:10.1371/journal.pone.0155164)
Supplement: S1 File — (DOCX) [file pone.0155164.s001.docx]

# **Supplementary Material**

## **Materials and methods**

### **Exploratory proteomics phase: sample preparation and LC-MS/MS analysis**

The UltiMate 3000 RSLCnano system hyphenated to a Q-Exactive mass spectrometer (Thermo Scientific) was used for protein identification and quantification. Fractions from high-pH reversed-phase separation were vacuum-dried, re-dissolved in loading phase A (2% ACN, 98% water and 0.05% TFA) and loaded onto a PepMap100 C18, 3 µm, 100 Å, 0.075 × 20 mm trap column. Peptides were separated on a PepMap RSLC C18, 2 µm, 100 Å, 0.075 × 150 mm analytical column by a gradient formed bymobile phase A (0.1% FA) and mobile phase B (80% ACN, 0.1% FA) running from 4% to 34% in 48 min and from 34% to 55% in 15 min of mobile phase B, at a flow rate of 0.3 µL/min at 40°C. The full MS/Top10 experimental setup was used. Positive- ion full-scan MS spectra (*m/z* 300-1800) were acquired using a 1×10^6^ AGC target at a resolution of 70,000 (at *m/z* 200). The lock mass of *m/z* 445.12003 was used for internal calibration. Precursors with a charge state ≥ 2 and a threshold intensity of 1×10^5^ were selected for HCD fragmentation, with an exclusion window of 60 s. The isolation window of 2 Da and normalized collision energy of 30 were used. Each HCD spectrum was acquired at a resolution of 35,000, with a 5×10^5^ AGC MSMS target and a maximum 120 ms injection time. Each fraction was analyzed twice the same way.

HCD spectra were searched in the Proteome Discoverer software (Thermo Scientific), using MASCOT (Matrix Science, London, UK) against the human UniProt protein database. The trypsin specificity was set at full and two missed cleavages were allowed. The mass tolerance was set at 10 ppm for precursors and at 20 mmu for fragment ions. Oxidized Met and Pro were set as dynamic modifications, while the thiomethylation of Cys, iTRAQ modification of Lys and peptide N-terminus were set as fixed modifications. A percolator was used for rescoring the MASCOT search results. Peptide to spectrum matches up to 1% FDR were reported. Only precursors with maximum 20% co-isolation, resulting in the identification of unique peptides, were used for quantification. The quantitative results were corrected on purity factors of iTRAQ labels and normalized by the median protein changes. Only proteins quantified with at least three peptides were evaluated.
