# Supplementary material for: Proteomic Analysis of Early Mid-Trimester Amniotic Fluid Does Not Predict Spontaneous Preterm Delivery
Source: PLoS One. 2016 May 23;11(5):e0155164. doi: 10.1371/journal.pone.0155164 (PMC4876998; doi:10.1371/journal.pone.0155164)
Supplement: S1 Table — The proteins are ranked according to the absolute value of log2 of average of 115/114 and 117/116 ratio. (DOCX) [file pone.0155164.s002.docx]

# **Supplementary Material**

## **Results**

### **Proteomics exploratory phase: LC-MS/MS analysis**

**S1 Table. The complete list of the 17 proteins that were reproducibly upregulated**

**where 115 and 117 represent the channels for the cases and 114 and 116 represent**

**the channels for the controls. The proteins are ranked according to the absolute value**

**of log2 of average of 115/114 and 117/116 ratio.**

**Accession Gene Description 115/114 117/116**

P02741 CRP C-reactive protein 2.27 2.30

P60174 TPI1 Triosephosphate isomerase 2.26 1.95

A8K7I4 CLCA1 Calcium-activated chloride 1.75 1.98

channel regulator 1

P40925 MDH1 Malate dehydrogenase, cytoplasmic 1.86 1.63

P01037 CST1 Cystatin-SN 1.46 1.51

Q6UWV6 ENPP7 Ectonucleotide pyrophosphatase/ 1.49 1.45

phosphodiesterase family member 7

P00915 CA1 Carbonic anhydrase 1 1.40 1.52

P02790 HPX Hemopexin 1.38 1.42

P48637 GSS Glutathione synthetase 1.38 1.41

Q96IY4 CPB2 Carboxypeptidase B2 1.35 1.37

O00187 MASP2 Mannan-binding lectin serine 1.32 1.33

protease 2

P17174 GOT1 Aspartate aminotransferase, 1.42 1.23

cytoplasmic

P62937 PPIA Peptidyl-prolyl cis-trans isomerase A 1.38 1.22

P08263 GSTA1 Glutathione S-transferase A1 1.36 1.22

P08697 SERPINF2 Alpha-2-antiplasmin 1.29 1.29

P00747 PLG Plasminogen 1.29 1.28

Q9Y6R7 FCGBP IgGFc-binding protein 1.30 1.24
