# Supplementary material for: Proteomic Analysis of Early Mid-Trimester Amniotic Fluid Does Not Predict Spontaneous Preterm Delivery
Source: PLoS One. 2016 May 23;11(5):e0155164. doi: 10.1371/journal.pone.0155164 (PMC4876998; doi:10.1371/journal.pone.0155164)
Supplement: S2 Table — The proteins are ranked according to the absolute value of log2 of average of 115/114 and 117/116 ratio. (DOCX) [file pone.0155164.s003.docx]

# **Supplementary Material**

## **Results**

### **Proteomics exploratory phase: LC-MS/MS analysis**

**S2 Table. The complete list of the 19 proteins that were downregulated where 115 and 117**

**represent the channels for the cases and 114 and 116 represent the channels for the controls.**

**The proteins are ranked according to the absolute value of log2 of average of 115/114 and**

**117/116 ratio.**

**Accession Gene Description 115/114 117/116**

P02042 HBD Hemoglobin subunit delta 0.56 0.52

P69905 HBA1 Hemoglobin subunit alpha 0.60 0.51

P68871 HBB Hemoglobin subunit beta 0.61 0.52

P09466 PAEP Glycodelin 0.61 0.59

P49913 CAMP Cathelicidin antimicrobial peptide 0.65 0.71

P00918 CA2 Carbonic anhydrase 2 0.65 0.75

O75015 FCGR3B Low affinity immunoglobulin gamma 0.67 0.74

Fc region receptor III-B

P69891 HBG1 Hemoglobin subunit gamma-1 0.72 0.71

Q16270 IGFBP7 Insulin-like growth factor-binding 0.69 0.75

protein 7

P08670 VIM Vimentin 0.72 0.72

P80188 LCN2 Neutrophil gelatinase-associated 0.76 0.70 lipocalin

P05109 S100A8 Protein S100-A8 0.70 0.77

P06702 S100A9 Protein S100-A9 0.70 0.78

P06744 GPI Glucose-6-phosphate isomerase 0.75 0.76

P05121 SERPINE1 Plasminogen activator inhibitor 1 0.78 0.74

P13796 LCP1 Plastin-2 0.77 0.77

P02679 FGG Fibrinogen gamma chain 0.79 0.77

P49747 COMP Cartilage oligomeric matrix protein 0.79 0.77

P10451 SPP1 Osteopontin 0.81 0.81
